# Effects of *Rhizobium* Species Living with the Dark Septate Endophytic Fungus *Veronaeopsis simplex* on Organic Substrate Utilization by the Host

**DOI:** 10.1264/jsme2.ME17144

**Published:** 2018-03-29

**Authors:** Yong Guo, Yuuto Matsuoka, Tomoyasu Nishizawa, Hiroyuki Ohta, Kazuhiko Narisawa

**Affiliations:** 1 Ibaraki University College of Agriculture Ibaraki, Japan, 3–21–1 Chuou, Ami, Ibaraki 300–0393 Japan; 2 Graduate School of Agriculture, Ibaraki University Ibaraki, Japan, 3–21–1 Chuou, Ami, Ibaraki 300–0393 Japan; 3 United Graduate School of Agricultural Science, Tokyo University of Agriculture and Technology 3–5–8 Saiwai-cho, Fuchu-shi, Tokyo 183–8509 Japan

**Keywords:** dark septate endophyte, *Veronaeopsis simplex*, endophyte-associated rhizobia, organic substrate utilization, tripartite symbiosis

## Abstract

Bacteria harbored in/on the hyphae of the dark septate endophyte, *Veronaeopsis simplex* Y34, were identified as a single *Rhizobium* species by molecular analyses of bacterial 16S rRNA genes, and were successfully isolated from the endophyte. The *Rhizobium*-cured fungus was prepared thoroughly by an antibiotic treatment, thereby allowing an examination of their effects on organic substrate utilization. Assays with Biolog^®^ FF microplates revealed that the respiration potential for 52.6% of the tested compounds were significantly different between *Rhizobium*-harboring and -cured fungal hosts, indicating that organic substrate utilization by *V. simplex* Y34 was significantly influenced by the presence of the associated *Rhizobium* sp. VsBac-Y9.

Plant-microbe interactions drive plant health and the biogeochemical cycle in terrestrial ecosystems (1, 6, 7). Plants provide important habitats and deliver photosynthates to their associated microorganisms (15). In return, mutualistic microbes promote host growth via improved mineral and nutrient uptake, enhanced tolerance against environmental stress, and/or deterring encroachment by phytopathogens (19, 26, 28, 33). These symbiotic interactions have been adequately evaluated in the bipartite relationships between plants and mycorrhizal fungi or nodule bacteria (14, 32); however, increasing evidence has revealed more complex associations among plants, endophytic fungi, and rhizobacteria since the discovery of hyphae-epigenous (epihyphal) and endohyphal bacteria in various plant-associated fungi (13, 34). Phylogenetically diverse bacteria have been identified inside and outside (*i.e.*, in/on) the hyphae of foliar *Ascomycota*, arbuscular mycorrhizal fungi (AMF), ectomycorrhizal basidiomycetes, and Sebacinalean endophytes. These bacteria have been shown to alter host morphology, sporulation, metabolite production, and other properties involved in symbiotic relationships with plants (2, 8, 27, 31). However, this tripartite symbiosis has rarely been reported for other widespread dark septate endophytes (DSE), which are defined as conidial or sterile ascomycetous fungi that live in symbiosis with certain plants and colonize plant root tissue intracellularly and intercellularly without any disease or typical mycorrhizal structures (17, 24). Due to the crucial importance of nutrient interchange between plants and endophytic fungi for maintaining their symbiotic relationship (15, 20), the mechanisms by which DSE-associated bacteria affect the metabolism of organic substrates by the host fungus need to be elucidated in order to assess the physiological and ecological roles of bacteria in tripartite symbioses. In our recent study, the DSE species, *Veronaeopsis simplex*, was found to be associated with bacterium-like cells (BLCs) on its hyphal surface in examinations of fluorescence microscopy images using a Live/Dead *Bac*Light kit (Khastini, unpublished data). The aims of the present study were to (i) identify *V. simplex*-associated bacteria using a molecular analysis of 16S rRNA genes, (ii) isolate bacteria harbored in fungal hyphae, (iii) remove bacteria from living fungi by an antibiotic treatment, and (iv) further examine the effects of DSE-associated bacteria on organic substrate utilization by the host through comparisons of respiratory activity and mycelial growth between bacteria-harboring and -cured *V. simplex* isolates on 95 carbon sources using the Biolog FF MicroPlate (BiOLOG, Hayward, CA, USA).

*V. simplex* was initially isolated from the litter below *Acacia karroo* in South Africa (isolate CBS 588.66) (3). In our previous study, two *V. simplex* isolates Y34 and IBAK45 (unpublished) were obtained in 2007 from the forest soil of Yaku Island and Ami, Japan, respectively, using the eggplant baiting method (18). These three isolates were used to identify DSE-associated bacteria. We removed loosely-associated bacteria (potentially contaminating bacteria) from the fungus using the modified van Tieghem method (29). The presence/absence of bacteria inside and outside of the hyphae of the original and van Tieghem-treated fungus was assessed microscopically by staining with a LIVE/DEAD *Bac*Light™ Bacterial Viability Kit (Thermo Fisher Scientific, Waltham, MA, USA) and by fluorescence *in situ* hybridization (FISH) using the universal bacterial probe EUB338, as previously described (25). The endobacterium-harboring fungus, *Mortierella elongata* FMR23-6 I-B1, was simultaneously observed as a positive control for confirming the presence of endohyphal bacteria (25, 29). Fluorescent BLCs were only detected on the hyphal surface of the original *V. simplex* Y34 and IBAK45, and not on that of CBS 588.66 or any van Tieghem-treated isolates by the LIVE/DEAD *Bac*Light Bacterial Viability Kit (Fig. 1) and 16S rRNA gene targeting FISH (Fig. S1).

In order to confirm BLC-free fungi, PCR amplification of the bacterial 16S rRNA gene was conducted using 0.1 μg of template DNA extracted from the hyphae of van Tieghem-treated isolates. Although BLC was not detected in microscopic observations, the targeted DNA band was obtained by PCR amplification for all van Tieghem treated-fungal isolates, suggesting that a very low population of bacteria harbored van Tieghem-treated fungi. In order to establish the number of bacterial species associated with the fungus, a terminal restriction fragment length polymorphism (T-RFLP) (29) analysis was performed using DNA extracted from the whole cell lysates of van Tieghem-treated fungi (Supplementary Methodology). T-RFLP profiles were composed of one major T-RF: a 189-base T-RF from the *Hae*III digest, a 338-base T-RF from the *Hha*I digest, and a 126-base T-RF from the *Msp*I digest (Fig. S2). The relative abundance of these major T-RFs ranged between 79.7% and 98.1% in each profile (Fig. S2). These results suggested that a single bacterial species was exclusively associated with the hyphae of *V. simplex* isolates.

In order to identify the fungus-associated bacterium exhibiting the major T-RF, clone libraries were constructed from the same DNA preparations mentioned above, as described in Supplementary Methodology. Since the detection of a single population was expected from T-RFLP profiling, only a few clone sequences were analyzed: 4 from *V. simplex* CBS 588.66, 7 from Y34, and 3 from IBAK45. Eleven of the 14 clone sequences showed 99.7–100% similarities to each other for the average length of 690 bp. BLAST searches with the NCBI database indicated that all bacterial sequences originating from the Y34 and IBAK45 isolates were closely related to *Rhizobium* sp. enrichment culture clone N312 with high similarity (99%). To the best of our knowledge, some intracellular bacteria in the genus *Rhizobium* were discovered in the hyphae of the endophytic Sebacinalean fungus *Piriformospora indica* (31) and diverse *Ascomycota* isolated as foliar endophytes of cupressaceous trees (4).

In an attempt to isolate the *V. simplex*-associated bacterium, aliquots of the filtered fungal homogenates of isolates Y34, IBAK45, and CBS 588.66 were separately spread on Difco™ nutrient broth (NB) agar plates and incubated at 30°C for 7 d. Bacterial colonies only appeared on the NB agar plate inoculated with the *V. simplex* Y34 homogenate. Since these colonies from the homogenate were very homogeneous, a single colony from the plate was subcultured repeatedly on an NB plate in order to assess its purity. The pure culture obtained, designated VsBac-Y9, was a Gram-negative rod-shaped bacterium. In order to establish whether this strain is a *V. simplex*-associated bacterium, a T-RFLP analysis of the 16S rRNA gene of strain VsBac-Y9 was performed, and showed combinations of a single T-RF (189-, 337-, and 126-base T-RFs from *Hae*III, *Hha*I, and *MspI* digestion). In the phylogenetic tree (Fig. 2), strain VsBac-Y9 was affiliated with the cluster composed of the above-described clone sequences and *Rhizobium* sp, enrichment clone N312. A BLAST search of its almost-complete 16S rRNA gene sequence (1387 bp) with the NCBI database showed that isolate VsBac-Y9 was extremely close to the *Rhizobium* isolates P7 and YIC4260, which were cultured from the rhizospheres of gramineous *Lolium multiflorum* (37) and leguminous *Sesbania cannabina* (21), respectively.

As shown in Fig. 2, the cluster composed of *V. simplex-*associated bacteria was close to *R. radiobacter* PABac-DSM identified as the endohyphal bacterium of *P. indica* (31). The most recent study of this endohyphal bacterium showed low numbers of *R. radiobacter* F4 (a subculture from *R. radiobacter* PABac-DSM) in axenically grown *P. indica* (long-term labcultured for 20 years), but high numbers in hyphae colonizing living plants or freshly re-isolated *P. indica* from plant roots (12), providing a potential explanation for why the population of associated bacteria was extremely small inside or outside the hyphae of *V. simplex* isolates (lab-cultured for 10 years). Furthermore, a phylogenetic analysis showed that these endophyte-associated bacteria were within the *Agrobacterium*/*Rhizobium* clade containing many phytopathogens and distant from the N-fixing symbiotic bacteria of leguminous plants, suggesting the necessity for further morphological and genomic characterizations to assign new positions for *Rhizobium* bacteria associated with endophytic fungi.

In order to assess the effects of *V. simplex*-associated rhizobia on organic substrate utilization by their host fungi, we eliminated bacteria present in the hyphae of van Tieghem-treated isolates using an antibiotic treatment (Supplementary Methodology). Antibiotic-treated hyphae were then grown on 1/2 CMMY agar to examine whether bacteria were present using PCR amplification of the partial 16S rRNA gene. The results obtained showed that only *V. simplex* Y34 was successfully cured of the associated *Rhizobium* spp. Therefore, only isolates of *Rhizobium*-harboring *V. simplex* Y34 and -cured Y34 were subsequently used to investigate the effects of rhizobia on organic substrate utilization by their fungal host *V. simplex* Y34 (Supplementary Methodology). The results demonstrated that the respiration potential for 50 out of 95 (52.6%) organic sources was significantly different between *Rhizobium-*associated and -cured isolates (Table 1, S1). *Rhizobium-*harboring *V. simplex* Y34 outperformed its cured isolate on 27 substrates including 9 saccharides, 7 saccharide derivatives, 3 amino acids and their derivatives, 3 carboxylic acids, 3 amides, 1 phosphorylated chemical, and 1 surfactant. In contrast, cured *V. simplex* Y34 was more active on 23 substrates than the *Rhizobium*-harboring isolate (*i.e.*, 9 carboxylic acids, 8 saccharides and their derivatives, 3 amino acids, 1 ester, 1 amide, and 1 brominated chemical). On the other hand, the growth of *Rhizobium*-harboring *V. simplex* Y34 was greater than that of the cured isolate across most organic compounds (Table S1). In addition, Jaccard dissimilarity indices suggested that the global substrate utilization of *V. simplex* Y34 was significantly influenced by the presence of associated *Rhizobium* spp. (Table 2).

An increasing number of studies have demonstrated that phylogenetically diverse bacteria associated with endophytic fungi critically influence host morphology, growth, sporulation, bioenergetic capacity, and metabolic products, which further affect the symbiotic capacity of the host with plants (23, 27, 35). The mechanisms by which endophyte-associated bacteria regulate the relationship between their fungal host and the specific symbiotic plant are of interest. Duponnois and Garbaye (1990) reported that ectomycorrhizal helper bacteria released citric and malic acids, which stimulated the growth of *Paxillus involutus* and metabolized self-toxins (*i.e.*, polyphenolic substances) produced by *P. involutus* (9). In addition, a recent study showed that the composition of the bacterial community associated with the saprotrophic fungus *Mucor hiemalis* shifted after an antibiotic treatment, which led to significant reductions in fungal secondary metabolites such as trifluorbenzene and butanol (30). Since these volatiles may improve the growth of certain rhizobacteria (*e.g.*, *Burkholderia* sp. AD024) (30), this finding suggests that fungal-associated bacteria affect the metabolic properties of a host, and these changes, in turn, influence the associated bacteria. Our comparative catabolism analysis revealed that *Rhizobium*-harboring *V. simplex* Y34 exhibited a preference for saccharides (*e.g.*, dextrin and glycogen) and saccharide derivatives (*e.g.*, arbutin, D-glucosamine, and N-acetyl-D-galactosamine), whereas cured isolate Y34 preferentially used carboxylic acids (*e.g.*, β-hydroxy-butyric acid, D-glucuronic acid, D-saccharic acid, D-malic acid, and sebacic acid). Based on these differences in organic substrate utilization, we speculated that *Rhizobium* spp. present in *V. simplex* may regulate their host fungi to effectively and selectively catabolize certain compounds released by symbiotic plants or contained in the rhizosphere, potentially by secreting growth factors for the host fungus (11, 27), detoxifying harmful waste, or metabolizing byproducts such as reactive oxygen species, which are known to accumulate from the catabolism of certain compounds (22, 36). Approximately 20–40% of the photosynthetic products fixed by a plant are released into the rhizosphere as root exudates and further utilized by root-associated bacteria and fungi (16), whereas fungal-bacterial interactions in this “hot-spot” may affect plant-microbe symbiotic relationships. Therefore, our results provide novel insights into the metabolism of organic substrates, which may ultimately contribute to our understanding of the ecological role of each partner in tripartite symbiosis. Future studies that directly assess how *Rhizobium* species induce changes in organic compound catabolism and affect the symbiotic performance of host fungi with certain plants are needed.

In conclusion, this study identified DSE-associated bacteria with *V. simplex* using a 16S rRNA gene targeting clone library analysis despite negative detection by microscopic observations. We also successfully eliminated intimately associated *Rhizobium* species from *V. simplex* isolate Y34 using an antibiotic treatment. The results obtained demonstrated the effects of DSE-associated *Rhizobium* spp. on organic substrate utilization by their host endophytic fungus *V. simplex* Y34 across 95 compounds including most sugars, amino acids, and carboxylic acids, nearly all of which are involved in plant biology (*e.g.*, global metabolism regulators: D-trehalose and *m*-inositol; metabolites involved in seed germination and root induction: dextrin, D-raffinose, L-asparagine, stachyose, and sucrose; structural components: D-mannose, L-alanine, L-arabinose and starch). To the best of our knowledge, this study is the first to have described the symbiotic bacteria associated with DSE and attempted to clarify the physiological and ecological roles of these bacteria in tripartite *V. simplex* symbiosis with plants by examining the effects of the associated *Rhizobium* bacteria on organic substrate utilization by their host fungus. In order to obtain a better understanding of plant-microbe symbiosis in natural and agroecological systems, future research is needed on the endophyte-associated bacteriome, bacterial localization, the mechanisms underlying interactions, and its importance for maintaining symbiotic relationships.

## Supplementary Material



## Figures and Tables

**Fig. 1:**
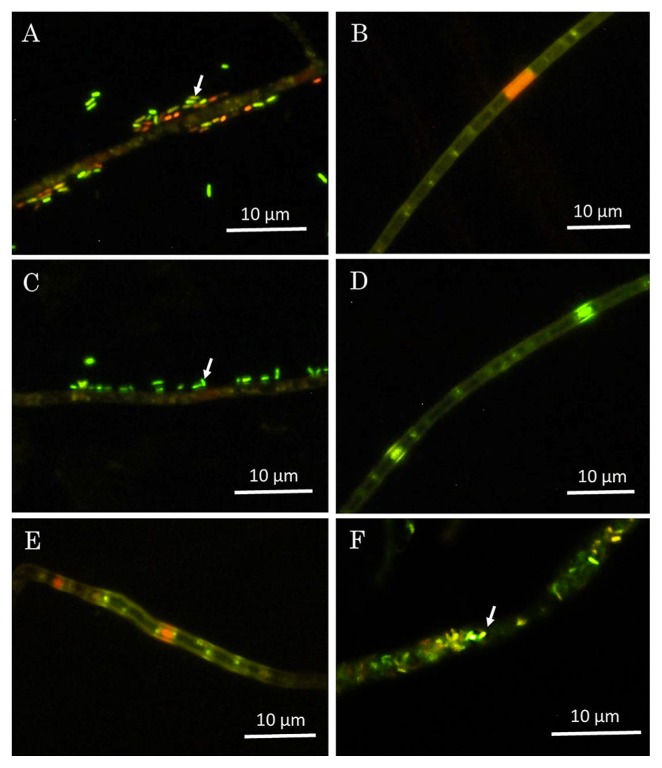
Fluorescence microscopic observations of fungal mycelia using a Live/Dead *Baclight* kit. A and B, original isolate and van Tieghem-treated isolate of *Veronaeopsis simplex* Y34, respectively; C and D, original isolate and van Tieghem-treated isolate of *V. simplex* IBAK45, respectively; E, original isolate of *V. simplex* strain CBS 588.66; F, original isolate of *Mortierella elongata* FMR23-6 I-B1. Arrows in A, C, and F indicate bacterium-like cells (BLCs) epihyphally or endohyphally associated with hyphae.

**Fig. 2:**
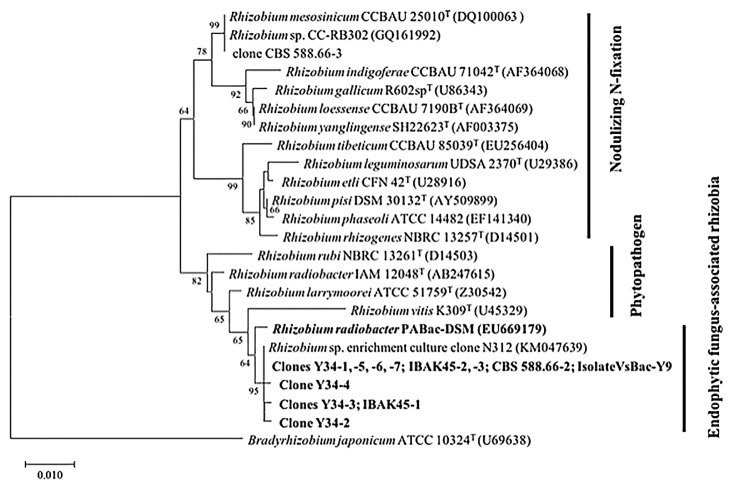
Neighbor-joining phylogenetic tree based on partial 16S rRNA genes (829 positions), showing the relationship between *Veronaeopsis simplex*-associated bacteria and the endohyphal *Rhizobium radiobacter* PABac-DSM of *Piriformospora indica*, and other species of the genus *Rhizobium*. Numbers at nodes are posterior probability values (%); values lower than 60% are not shown. Horizontal lines show genetic distances, which are supported by values estimated with 1,000 bootstrap replicates. The scale bar indicates the number of substitutions per nucleotide position. *Bradyrhizobium japonicum* ATCC 10324^T^ is used as the outgroup.

**Table 1:** Comparison of significantly outperforming carbon sources by *Rhizobium*-harboring and -cured *Veronaeopsis simplex* Y34

Category of organic substrate	Significantly outperformed organic substrate^a^	

	*Rhizobium*-harboring *V. simplex* Y34	*Rhizobium*-cured *V. simplex* Y34
Monosaccharides (12)^b^	D-psicose; (1)^c^	D-arabinose, D-fructose, D-ribose, **D-xylose**^d^; (4)^c^
Disaccharides (10)	α-D-lactose, D-melibiose, D-trehalose, lactulose; (4)	—
Trisaccharides (3)	—	D-raffinose; (1)
Tetrasaccharide (1)	—	Stachyose; (1)
Oligosaccharide (1)	Dextrin; (1)	—
Cyclic oligosaccharides (2)	β-cyclodextrin; (1)	—
Nucleosides (2)	Adenosine; (1)	Uridine; (1)
Polysaccharide (1)	Glycogen; (1)	—
Sugar alcohols (9)	—	Glycerol; (1)
Methyl sugars (5)	β-methyl-D-galactoside; (1)	—
Alcoholic β-glucoside (1)	Salicin; (1)	—
Glycoside (1)	**Arbutin**^d^; (1)	—
Misc. carbohydrate (1)	Sedoheptulosan; (1)	—
Amino sugars (4)	D-glucosamine, N-acetyl-D-galactosamine, N-acetyl-D-glucosamine; (3)	—
Amino acids (14)	L-alanine, L-asparagine; (2)	L-threonine, **N-acetyl-L-glutamic acid**, **L-pyroglutamic acid**; (3)
Amino acid derivative (1)	Amygdalin; (1)	—
Carboxylic acids (16)	α-ketoglutaric acid, L-lactic acid, γ-hydroxy-butyric acid; (3)	2-keto-D-gluconic Acid, β-hydroxy-butyric acid, D-glucuronic acid, D-galacturonic acid, D-gluconic acid, D-saccharic acid, **D-malic acid**, fumaric acid, sebacic acid; (9)
Esters (2)	—	Succinic acid monomethyl ester; (1)
Amides (5)	**Alaninamide**, Glucuronamide, Putrescine; (3)	**Succinamic acid**; (1)
Phosphorylated chemicals (2)	Glucose-1-Phosphate; (1)	—
Brominated chemical (1)	—	Bromosuccinic acid; (1)
Surfactant (1)	Tween^®^ 80; (1)	—
Total (95)	27^e^	23^e^

a Significant differences in the respiration (OD 490 nm) of each organic substrate performed between *Rhizobium*-harboring and -cured *Veronaeopsis simplex* Y34 were examined by a one-way ANOVA with an FDR-adjusted *P*-value (*P*<0.05) (5)

b Amount of organic substrates for each category

c Amount of significantly outperformed organic substrates for each category by *Rhizobium*-harboring or -cured *V. simplex* Y34

d Compounds printed in Bold indicate a significant difference in respiration with FDR-adjusted *P*<0.001

e Total amount of significantly outperformed organic substrates within a BIOLOG FF Microplate by *Rhizobium*-harboring and -cured *V. simplex* Y34, respectively

**Table 2:** Global dissimilarities in the profile of carbon source utilization between *Rhizobium*-harboring and -cured *Veronaeopsis simplex* Y34

Sample		*Rhizobium*-harboring *V. simplex* Y34			*Rhizobium*-cured *V. simplex* Y34		
	
		Replicate 1	Replicate 2	Replicate 3	Replicate 1	Replicate 2	Replicate 3
*Rhizobium*-harboring *V. simplex* Y34	Replicate 1	0.0000^a^					
	Replicate 2	0.0574	0.0000				
	Replicate 3	0.1377	0.1092	0.0000			
*Rhizobium*-cured *V. simplex* Y34	Replicate 1	**0.2635**	**0.2593**	**0.2903**	0.0000		
	Replicate 2	**0.2639**	**0.2610**	**0.2917**	0.0595	0.0000	
	Replicate 3	**0.2732**	**0.2595**	**0.2830**	0.0895	0.0764	0.0000

a This value was calculated by the Jaccard classic dissimilarity index (10)
