# Supplementary material for: Effects of Rhizobium Species Living with the Dark Septate Endophytic Fungus Veronaeopsis simplex on Organic Substrate Utilization by the Host
Source: Microbes Environ. 2018 Mar 29;33(1):102–6. doi: 10.1264/jsme2.ME17144 (PMC5877336; doi:10.1264/jsme2.ME17144)
Supplement: Supplementary file 1 [file 33_102_s1.pdf]

Supplementary Fig. S1. Fluorescence microscopic observations of FISH-stained fungal mycelia with the universal bacteria probe EUB338 (Red). A and B, original isolate and van Tieghem-treated isolate of *Veronaeopsis simplex* Y34, respectively; C and D, original isolate and van Tieghem-treated isolate of *V. simplex* IBAK45, respectively; E, original isolate of *V. simplex* CBS 588.66; F, original isolate of *Mortierella elongata* FMR23-6 I-B1. Arrows in A, C, and F indicate BLCs epihyphally or endohyphally associated with hyphae.

Supplementary Fig. S2. Bacterial 16S rRNA gene-targeting T-RFLP profiles of van Tieghem-treated *Veronaeopsis simplex* isolates indicate the predominance of single species in the bacterial community associated with *V. simplex* isolates. *Hae*III, *Hha*I, and *Msp*I were used for digestion.

Supplementary Information

Methodology

Fungal isolates

In this study, we used *Veronaeopsis simplex* CBS 588.66 isolated from the litter below *Acacia karroo* in South Africa; *V. simplex* Y34 isolated from forest soil in Yakushima, Kakoshima, and *V. simplex* IBAK45 isolated from forest soil in Ami, Ibaraki Japan. All these isolates were incubated on half-strength cornmeal-malt-yeast (1/2 CMMY) agar plate at 23°C.

Preliminary treatment and microscopic observation

We firstly removed loosely-associated bacteria with *V. simplex* using a modified van Tieghem method, as described by Sato et al., 2010 (7). The bacterial presence/absence inside and outside the hyphae of original and van Tieghem-treated *V. simplex* isolates was confirmed by staining using a LIVE/DEAD *Bac*Light^{TM} Bacterial Viability Kit (Thermo Fisher Scientific, Waltham, MA, USA) and observation by fluorescence *in situ* hybridization (FISH) with a universal bacterial probe EUB338, as previously described (3, 5). At the same time, an endobacteria-harboring fungus *Mortieralla elongata* FMR23-6 I-B1 was observed as a positive control for confirming the presence of endohyphal bacteria (5). Microscopic observation was conducted under blue and green light using a fluorescence microscope (BX51, Olympus, Tokyo, Japan) equipped with a complementary metal-oxide-semiconductor (CMOS) camera (Xi7, Canon, Tokyo, Japan).

DNA extraction, PCR amplification, T-RFLP, and clone library construction

The *V. simplex* isolates treated by the van Tieghem method were incubated on 1/2 CMMY agar for 6 weeks at 23°C. DNA was extracted from the recovered mycelia using a PrepMan® Ultra Sample Preparation Reagent (Thermo Fisher Scientific, Waltham, MA, USA) with a minor modification. PCR amplification of bacterial 16S rRNA genes for T-RFLP analysis was performed in a 30 μL reaction mixture containing 0.1 μg of template DNA, 5 pmol of primers (Qarc-10F and 926R), 1 × Ex buffer, 75 pmol of dNTPs, and 1.5 U of Ex *Taq* polymerase (Takara Bio, Shiga, Japan) in a thermal cycler (Bio-Rad, Hercules, CA, USA) using the following thermal conditions: 5 min at 95°C, then 25 cycles of 95°C for 30 s, 54°C for 45 s, and 72°C for 90 s (7). The fluorescently labeled PCR products were purified using a Qiagen PCR Purification Kit (Qiagen, Hilden, Germany), and eluted in a final volume of 40 μl. Aliquots (10 μl) of the purified 16S rRNA gene products were separately digested with 1 U of *Hae*III, *Hha*I, and *Msp*I (Takara Bio) for 2 h, according to the manufacturer's instructions. The fragments were purified using the Qiagen Gel Extraction Kit. The precise lengths of terminal restriction fragments (T-RFs) were determined using a 3130*xl* PE Applied Biosystems Automated DNA Sequencer (Applied Biosystems, Foster City, CA, USA) according to the method of Nishizawa et al. 2008 (4). The T-RFLP data were imported into GeneMapper software (version 3.7, Applied Biosystems). The peaks

were identified using 30 as the peak height cut-off value and 30–700 bases as the size range. The raw T-RFLP data sets were uploaded to T-REX software (2), which was used to denoise (one standard deviation using peak height) and align peaks (clustering threshold of 0.5, at most one peak per profile), and create a data matrix (samples vs. aligned T-RFs). The values in the data matrix represented the relative abundance of the T-RFs.

On the other hand, the un-fluorescently labeled PCR products amplified using primer set of 10F-926R were purified using a Qiagen PCR purification Kit (Qiagen, Hilden, Germany), then ligated into a pGEM-T-easy vector (Promega, Madison, WI, USA) and used to transform into *Escherichia coli* DH5α competent cells (TOYOBO, Osaka, Japan). The transformants were screened as described previously (5). Clone sequences were determined using a Sanger sequencer (3130*xl* Applied Biosystems Automated DNA Sequencer). The DNA sequences were assembled and trimmed using GENETYX-MAC (version 18, Software Development, Tokyo, Japan). The clone sequences were deposited in NCBI/DDBJ/EMBL with accession No. MF928742-MF928755.

Isolation of the V. simplex*-associated bacteria and identification of the bacterial isolate VsBac-Y9*

Isolation of the *V. simplex*-associated bacteria was performed according to a previously described method (3). In brief, the *V. simplex* isolates Y34, IBAK45 and CBS 588.66 were cultivated on 1/2 CMMY agar plates for 7 days at 23°C. Cultivated fungal mycelia were homogenized by sterilized stainless crusher on a shaking mixer for 5 min and centrifuged at 1800 × *g* for 10 min. The supernatant was filtered through 8-µm- and 3-µm-pore membrane filters to remove fragmented hyphae and sporangiospores. Aliquots of the filtered suspension were spread on Difico^{TM} nutrient broth agar and incubated for 7 days at 30°C. The bacterial growth was only confirmed on the NB agar plate inoculated with the aliquots of isolate Y34. After a purification step, a single colony was transferred onto a fresh NB agar plate, and the isolate was named as 'VsBac-Y9'. Genomic DNA of VsBac-Y9 was extracted using a lysozyme buffer method, and a most-complete 16S rRNA gene sequence was obtained by a PCR-based protocol using the genomic DNA as template (5). The sequence was determined and analyzed as the above-described methods. The sequences were deposited in NCBI/DDBJ/EMBL with accession No. MG251442. In addition, the partial 16S rRNA gene of VsBac-Y9 was analyzed by 16S rRNA gene-targeting T-RFLP to deduce the abundance of VsBac-Y9 in the bacterial community associated with the hyphae of *V. simplex* isolates, according to above mentioned method.

Phylogenetic analysis

Determined sequences of 16S rRNA gene clones were compared with similar nucleotide sequences retrieved from the NCBI/DDBJ/EMBL databases using the BLAST program. Multiple alignments with/without clone sequences were performed with Molecular Evolutionary Genetics Analysis (MEGA ver. 7.0.26). Evolutionary distances were calculated by Kimura 2-parameter method, and neighbor-joining trees were constructed with 1000-replicate bootstrap test.

Preparation of axenic fungal culture and Biolog fiamentous fungus (FF) microplate assays

For the *V. simplex* isolates associated with *Rhizobium* spp., we eliminated the bacteria harbored in the hyphae by antibiotic treatment of mycelia in a mix solution of 50 µg mL^{-1} ciprofloxacin, 50 µg mL^{-1} kanamycin, 100 µg mL^{-1} ampicillin, and 100 µg mL^{-1} chloramphenicol, for 24 hours at 23°C. The treated mycelia were then grown on 1/2 CMMY agar for 7 days at 23°C to check whether the bacteria were present, by PCR amplification of 16S rRNA gene as described above. Only van Tieghem-treated Y34 (*Rhizobium*-harboring *V. simplex* Y34) was cured of the associated bacteria, and defined as cured *V. simplex* Y34. The *Rhizobium*-harboring and -cured *V. simplex* Y34 were used to examine organic substrate utilization using a Biolog FF MicroPlate (BiOLOG, Hayward, CA, USA), as in previous study (8) with a minor modification. In brief, *V. simplex* Y34 associated with/without *Rhizobium* species were pre-incubated on a 1/2 CMMY agar covered with a piece of sterile cellophane for 7 days at 23°C, then the mycelia grown on the cellophane were recovered, avoiding the agar itself, and transferred into a 75 ml sterile blender containing 20 ml of BioLOG FF-IF broth to prepare a suspension of hyphal fragments. After centrifuging at 3000 g for 5 min, the pellet was recovered and suspended in new 20 ml of fresh FF-IF broth. The final inoculum with an absorbance (OD600 nm) of 0.2 were transferred to the BioLOG FF microplate (100 µL of inoculum in each well) in triplicate, and incubated at 23°C for 7 days. The relative respiration for each carbon source was measured by absorbance at 490 nm (OD490 nm) on the 5th day, and examined by one-way ANOVA with an FDR-corrected p-value (1) at RStudio v1.0.136 (R v3.4.0). The dissimilarity of global carbon source utilization between the *Rhizobium*-harboring and -cured Y34 were calculated by Jaccard classic dissimilarity indices using RStudio with the vegan package (6). At the same time, the growth of hyphae in each well was evaluated by visual observation.

References

1. Benjamini Y., Y. Hochberg. 1995. Controlling the false discovery rate: a practical and powerful approach to multiple testing. Journal of the royal statistical society Series B (Methodological):289-300.
2. Culman S.W., R. Bukowski, H.G. Gauch, H. Cadillo-Quiroz, D.H. Buckley. 2009. T-REX: software for the processing and analysis of T-RFLP data. BMC Bioinformatics 10:171.
3. Kai K., K. Furuyabu, A. Tani, H. Hayashi. 2012. Production of the Quorum‐Sensing Molecules N‐Acylhomoserine Lactones by Endobacteria Associated with *Mortierella alpina* A‐178. Chembiochem 13:1776-1784.
4. Nishizawa T., M. Komatsuzaki, N. Kaneko, H. Ohta. 2008. Archaeal diversity of upland rice field soils assessed by the terminal restriction fragment length polymorphism method combined with real time quantitative-PCR and a clone library analysis. Microbes and Environments 23:237-243.
5. Ohshima S., Y. Sato, R. Fujimura, Y. Takashima, M. Hamada, T. Nishizawa, et al. 2016. *Mycoavidus cysteinexigens* gen. nov., sp. nov., an endohyphal bacterium isolated from a soil isolate of the fungus

Mortierella elongata. Int J Syst Evol Microbiol 66:2052-7.

6. Okanen J., F.G. Blanchet, M. Frendly, R. Kindt, P.R. Legendre, D. McGlinn, et al. 2017. Vegan: community ecology package. https://cran.r-project.org/web/packages/vegan/index.html.
7. Sato Y., K. Narisawa, K. Tsuruta, M. Umezu, T. Nishizawa, K. Tanaka, et al. 2010. Detection of *Betaproteobacteria* inside the mycelium of the fungus *Mortierella elongata*. Microbes and Environments 25:321-324.
8. Singh M.P. 2009. Application of Biolog FF MicroPlate for substrate utilization and metabolite profiling of closely related fungi. J Microbiol Methods 77:102-8.

Supplementary Table S1. Comparison of absorbance at 490 nm for assessing the respiration and proliferation of hyphae between *Rhizobium*- harboring and -cured *Veronaeopsis simplex* Y34

		Rhizobium- harboring *V. simplex* Y34			*Rhizobium*- cured *V. simplex* Y34			One-way ANOVA	
Organic substrate	Category	Mean Abs@490nm	S.D.	Proliferation[a]	Mean Abs@490nm	S.D.	Proliferation[a]	Adjusted *p*-value by BH (FDR)	Mark[b]
α-D-Glucose	Monosaccharide	1.48	0.05	nd, nd, nd	1.52	0.09	w, w, nd	0.603	
D-Arabinose	Monosaccharide	0.43	0.01	nd, nd, nd	**0.49**	0.02	w, w, w	0.013	*
D-Fructose	Monosaccharide	1.16	0.11	+, +, +	**1.50**	0.12	w, w, w	0.048	*
D-Galactose	Monosaccharide	0.67	0.08	+, +, +	0.59	0.06	+, +, +	0.282	
D-Mannose	Monosaccharide	1.28	0.06	+, +, +	1.64	0.24	w, w, w	0.103	
D-Psicose	Monosaccharide	**0.69**	0.10	w. +, +	0.37	0.01	+, +, +	0.017	*
D-Ribose	Monosaccharide	0.70	0.03	nd, nd, nd	**0.98**	0.03	nd, nd, nd	0.003	**
D-Tagatose	Monosaccharide	0.45	0.09	+, +, +	0.62	0.03	+, +, +	0.075	
D-Xylose	Monosaccharide	0.64	0.05	+, +, +	**1.22**	0.01	w, w, w	0.001	***
L-Arabinose	Monosaccharide	0.62	0.04	+, +, +	0.65	0.01	+, +, +	0.362	
L-Fucose	Monosaccharide	0.39	0.04	+, +, +	0.39	0.02	+, +, +	0.967	
L-Sorbose	Monosaccharide	0.65	0.12	+, +, +	0.47	0.03	+, +, +	0.110	
α-D-Lactose	Disaccharide	**0.55**	0.09	+, +, +	0.36	0.02	+, +, +	0.042	*
D-Cellobiose	Disaccharide	1.30	0.12	w, w, w,	1.09	0.12	nd, nd, nd	0.140	
D-Melibiose	Disaccharide	**1.30**	0.02	nd, nd, nd	0.98	0.08	nd, nd, nd	0.009	**
D-Trehalose	Disaccharide	**1.50**	0.07	+, +, +	1.12	0.05	+, +, +	0.007	**
Gentiobiose	Disaccharide	1.35	0.10	+, +, +	1.25	0.09	+, +, +	0.310	
Palatinose (isomaltulose)	Disaccharide	1.35	0.10	+, +, +	1.62	0.16	w, w, +	0.097	
Lactulose	Disaccharide	**0.54**	0.08	+, +, +	0.36	0.00	+, +, +	0.042	*
Maltose	Disaccharide	1.59	0.09	+, +, +	1.75	0.05	w, w, w	0.084	
Sucrose	Disaccharide	1.28	0.10	+, +, +	1.54	0.18	+, +, +	0.129	
Turanose	Disaccharide	1.34	0.03	+, +, +	1.33	0.05	+, +, +	0.948	
D-Melezitose	Trisaccharide	1.45	0.05	+, +, +	1.58	0.31	+, +, +	0.553	
D-Raffinose	Trisaccharide	0.96	0.08	+, +, +	**1.41**	0.06	+, +, +	0.006	**
Maltotriose	Trisaccharide	1.50	0.03	+, +, +	1.79	0.15	+, +, +	0.051	
Stachyose	Tetrasaccharide	0.84	0.12	+, +, +	**1.13**	0.01	+, +, +	0.036	*
Dextrin	Oligosaccharide	**1.52**	0.07	w, w, w,	1.08	0.02	w, w, w	0.003	**
α-Cyclodextrin	Cyclic oligosaccharide	0.46	0.04	nd, nd, nd	0.43	0.03	nd, nd, nd	0.361	
β-Cyclodextrin	Cyclic oligosaccharide	**0.38**	0.02	nd, nd, nd	0.27	0.01	nd, nd, nd	0.005	**
Adenosine	Nucleoside	**0.87**	0.15	w, w, w,	0.33	0.01	nd, nd, nd	0.010	**
Uridine	Nucleoside	0.37	0.04	w, w, w,	**0.57**	0.03	nd, nd, nd	0.009	**
Glycogen	Polysaccharide	**1.28**	0.12	w, w, w,	0.55	0.05	nd, nd, nd	0.004	**
D-Arabitol	Sugar alcohol	0.56	0.07	+, +, +	0.51	0.02	+, +, +	0.358	
D-Mannitol	Sugar alcohol	0.91	0.07	+, +, +	0.90	0.05	w, w, w	0.946	
D-Sorbitol	Sugar alcohol	0.83	0.17	+, +, +	0.82	0.08	+, +, +	0.967	
Glycerol	Sugar alcohol	0.52	0.06	+, +, +	**0.82**	0.03	+, +, +	0.007	**
i-Erythritol	Sugar alcohol	0.85	0.03	+, +, +	0.81	0.04	+, +, +	0.222	
m-Inositol	Sugar alcohol	0.66	0.08	+, +, +	0.53	0.01	+, +, +	0.079	
Maltitol	Sugar alcohol	1.30	0.04	nd, nd, nd	1.34	0.10	nd, nd, nd	0.635	
Adonitol (ribitol)	Sugar alcohol	0.53	0.05	+, +, +	0.44	0.01	+, +, +	0.066	
Xylitol	Sugar alcohol	0.59	0.14	+, +, +	0.34	0.02	+, +, +	0.066	
L-Rhamnose	Methyl sugar	0.98	0.19	+, +, +	0.99	0.07	+, +, +	0.980	
α-Methyl-D-galactoside	Methyl sugar	0.53	0.13	nd, nd, nd	0.32	0.03	nd, nd, nd	0.084	
β-Methyl-D-galactoside	Methyl sugar	**0.57**	0.10	nd, nd, nd	0.36	0.04	nd, nd, nd	0.048	*
α-Methyl-D-glucoside	Methyl sugar	0.89	0.11	+, +, +	0.88	0.02	+, +, +	0.946	
β-Methyl-D-glucoside	Methyl sugar	0.88	0.13	+, +, +	0.68	0.02	+, +, +	0.085	
Salicin	Alcoholic β-glucoside	**0.47**	0.01	+, +, +	0.39	0.01	nd, w, w	0.003	**
Arbutin	Glycoside	**1.59**	0.12	+, +, +	0.36	0.03	w, +, w	0.001	***
Sedoheptulosan	Misc.carbohydrate	**0.56**	0.10	nd, nd, nd	0.32	0.02	nd, nd, nd	0.039	*
D-Glucosamine	Amino sugar	**0.85**	0.08	w, w, +	0.40	0.02	w, w, w	0.005	**
N-Acetyl-D-galactosamine	Amino sugar	**1.31**	0.04	nd, nd, w	1.06	0.05	nd, nd, nd	0.009	**
N-Acetyl-D-glucosamine	Amino sugar	**1.55**	0.10	w, w, w,	1.26	0.01	w, w, +	0.020	*
N-Acetyl-D-mannosamine	Amino sugar	0.46	0.15	w, w, w	0.32	0.02	nd, nd, nd	0.239	
L-Alanine	Amino acid	**1.45**	0.01	w, w, w,	1.05	0.06	nd, nd, nd	0.003	**
L-Proline	Amino acid	1.44	0.03	w, +, +	1.37	0.08	nd, nd, nd	0.254	
L-Serine	Amino acid	1.28	0.05	w, w, w,	1.24	0.07	nd, nd, nd	0.542	
L-Threonine	Amino acid	0.31	0.02	w, w, w,	**0.69**	0.06	w, nd, nd	0.004	**
L-Aspartic acid	Amino acid	1.20	0.10	w, w, w,	1.05	0.05	nd, nd, nd	0.113	
L-Glutamic acid	Amino acid	1.43	0.05	w, w, w,	1.39	0.10	nd, nd, nd	0.618	
N-Acetly-L-glutamic acid	Amino acid	0.65	0.06	nd, w, w	**1.49**	0.06	nd, nd, nd	0.001	***
Glycyl-L-glutamic acid	Amino acid	1.28	0.02	w, w, w,	1.28	0.08	nd, nd, nd	0.990	
L-Pyroglutamic acid	Amino acid	0.39	0.04	w, nd, w	**1.53**	0.08	nd, nd, nd	0.001	***
γ-Amino-butyric acid	Amino acid	0.53	0.03	w, +, +	0.47	0.01	w, w, w	0.089	
L-Asparagine	Amino acid	**1.41**	0.05	w, w, w,	1.27	0.02	nd, nd, nd	0.024	*
L-Phenylalanine	Amino acid	1.13	0.18	w, nd, w	1.41	0.06	nd, nd, nd	0.089	
L-Ornithine	Amino acid	1.12	0.13	w, w, w,	1.00	0.03	nd, nd, nd	0.249	
L-Alanyl-glycine	Amino acid	1.30	0.01	w, w, w,	1.21	0.06	nd, nd, nd	0.107	
Amygdalin	Amino acid derivative	**0.69**	0.10	w, w, w	0.35	0.01	w, w, w	0.011	*
2-Keto-D-gluconic acid	Carboxylic acid	0.52	0.09	nd, nd, nd	**0.83**	0.00	nd, nd, nd	0.013	*
α-Keto-glutaric acid	Carboxylic acid	**1.68**	0.02	nd, nd, nd	1.51	0.06	nd, nd, nd	0.024	*
β-Hydroxy-butyric acid	Carboxylic acid	0.60	0.05	+, +, nd	**1.06**	0.08	+, +, +	0.006	**
D-Glucuronic acid	Carboxylic acid	0.45	0.05	nd, w, w	**0.97**	0.06	nd, nd, nd	0.003	**
D-Galacturonic acid	Carboxylic acid	0.42	0.02	nd, nd, nd	**0.60**	0.03	nd, nd, nd	0.006	**
D-Gluconic acid	Carboxylic acid	0.54	0.10	w, w, w,	**0.83**	0.02	nd, nd, nd	0.019	*
D-Saccharic acid	Carboxylic acid	0.57	0.09	w, w, w,	**1.12**	0.09	w, w, w	0.006	**
D-Malic acid	Carboxylic acid	0.53	0.06	w, w, w,	**1.33**	0.05	nd, nd, nd	0.001	***
L-Malic acid	Carboxylic acid	1.32	0.03	w, w, w,	1.36	0.02	nd, nd, nd	0.192	
L-Lactic acid	Carboxylic acid	**1.29**	0.06	w, w, w,	0.92	0.05	w, w, w	0.006	**
γ-Hydroxy-butyric acid	Carboxylic acid	**1.18**	0.16	w, w,+	0.44	0.01	w, +, +	0.006	**
Fumaric acid	Carboxylic acid	1.36	0.04	+, +, +	**1.53**	0.07	w, w, w	0.041	*
p-Hydroxyphenyl-acetic acid	Carboxylic acid	0.71	0.55	+, +, +	1.40	0.12	w, w, w	0.140	
Quinic acid	Carboxylic acid	1.60	0.11	w, w, w,	1.68	0.08	w, nd, nd	0.401	
Sebacic acid	Carboxylic acid	0.56	0.02	w, w, w,	**0.89**	0.06	w, w, nd	0.005	**
Succinic acid	Carboxylic acid	1.29	0.02	nd, w, w	1.18	0.05	nd, nd, nd	0.056	
D-Lactic acid methyl ester	Ester	0.40	0.03	nd, nd, nd	0.60	0.16	nd, nd, nd	0.128	
Succinic acid mono-methyl ester	Ester	0.45	0.06	w, w, w,	**0.92**	0.22	nd, w, w	0.048	*
Alaninamide	Amide	**1.41**	0.03	nd, nd, w	0.43	0.04	nd, nd, nd	0.000	***
Glucuronamide	Amide	**0.47**	0.08	nd, nd, nd	0.27	0.00	nd, nd, nd	0.024	*
Succinamic acid	Amide	0.49	0.00	nd, w, w	**1.45**	0.08	nd, w, w	0.001	***
2-Amino ethanol	Amide	0.41	0.03	w, w, w,	0.38	0.02	nd, nd, nd	0.222	
Putrescine	Amide	**0.62**	0.12	w, w, w,	0.36	0.03	w, nd, nd	0.048	*
Glucose-1-phosphate	Phosphorylated chemical	**0.99**	0.15	w, w, w,	0.37	0.03	nd, nd, nd	0.009	**
Adenosine-5'-monophosphate	Phosphorylated chemical	0.61	0.08	w, w, w,	0.48	0.12	nd, nd, nd	0.239	
Bromosuccinic acid	Brominated chemical	0.78	0.03	w, w, w,	**1.03**	0.06	w, w, w	0.010	*
Tween 80	Surfactant	**0.84**	0.04	w, w, w	0.63	0.00	w, w, w	0.004	**
**Water**	**Blank**	**0.52**	0.07	nd, nd, nd	0.31	0.01	nd, nd, nd	0.021	*

[a]+, obvious growth; w, week growth; nd, not detected growth.

[b]*, 0.01≤p<0.05; **, 0.001≤p<0.01; ***, p<0.001

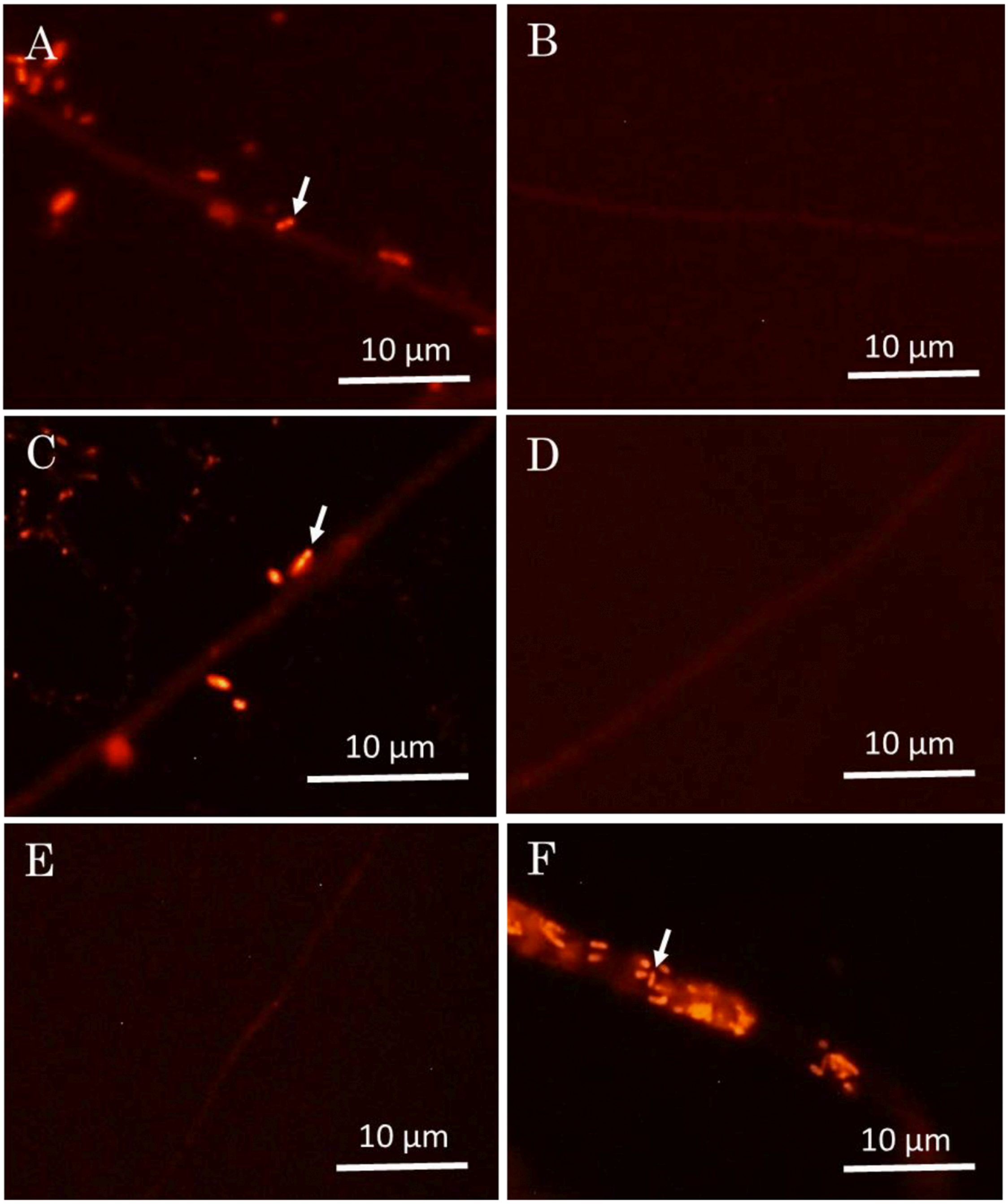

Supplementary Fig. S1. Fluorescence microscopic observations of FISH-stained fungal mycelia with universal bacteria probe EUB338 (Red). A and B, original isolate and van Tieghem-treated isolate of *Veronaeopsis simplex* Y34, respectively; C and D, original isolate and van Tieghem-treated isolate of *V. simplex* IBAK45, respectively; E, original isolate of *V. simplex* CBS 588.66; F, original isolate of *Mortierella elongata* FMR23-6 I-B1. Arrows in A, C, and F indicate BLCs epihyphally or endohyphally associated with the hyphae.

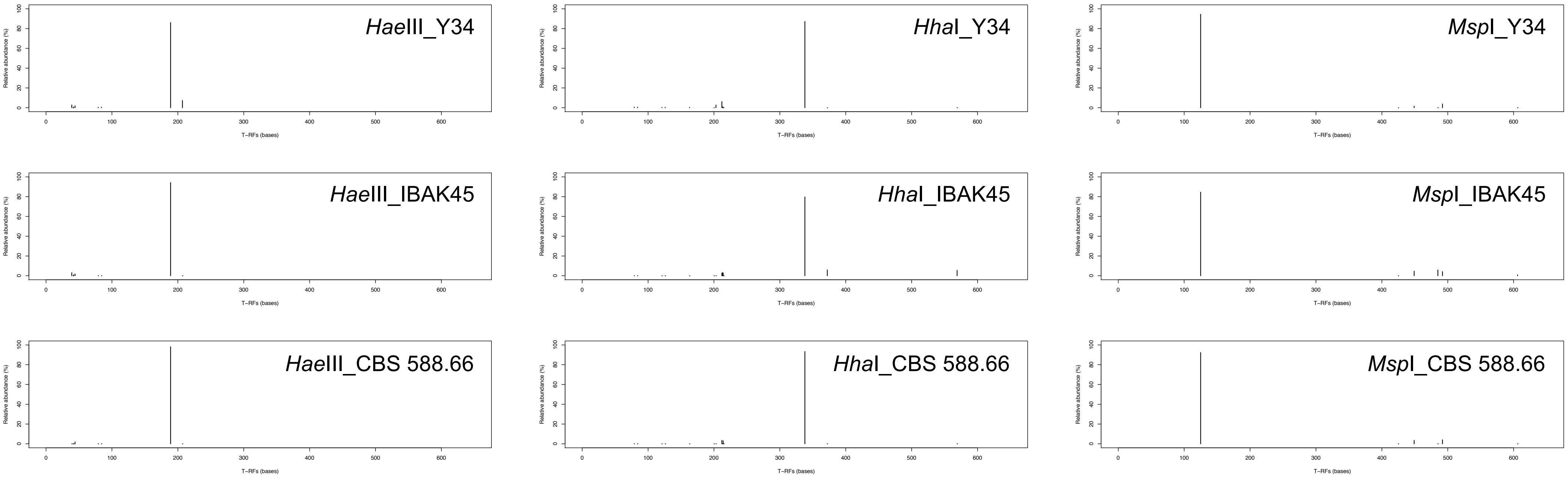

Supplementary Fig. S2. Bacterial 16S rRNA gene-targeting T-RFLP profiles of van Tieghem-treated *Veronaeopsis simplex* isolates indicate a predominance of single species in the bacterial community intimately associated with *V. simplex* isolates. *Hae*III, *Hha*I and *Msp*I were used as the digestion.
